# The Effects of a Comprehensive Rehabilitation Program of Alzheimer’s Disease in a Hospital Setting

**DOI:** 10.1155/2007/782959

**Published:** 2007-02-12

**Authors:** Alberto Raggi, Sandro Iannaccone, Alessandra Marcone, Valeria Ginex, Paola Ortelli, Alessandro Nonis, Maria Cristina Giusti, Stefano F. Cappa

**Affiliations:** ^1^Department of Psychology and Neuroscience, Vita-Salute San Raffaele University, MilanItaly; ^2^Division of Neurology, San Raffaele Turro Hospital, MilanItaly; ^3^USSB—University Statistics Centre for Biomedical Sciences, Vita-Salute San Raffaele University, MilanItaly

**Keywords:** Alzheimer’s Disease (AD), cognitive-behavioural rehabilitation, neuropsychiatric symptoms, Reality Orientation Therapy (ROT)

## Abstract

*Introduction.* The evidence for the clinical effectiveness of cognitive rehabilitation in patients with Alzheimer’s Disease (AD) is debated. Therefore it is important to collect more evidence about the outcome of non-pharmacological therapy of dementia. *Material and Methods.* We report data concerning the rehabilitation of 50 patients with probable AD admitted during a 17-month period in a specialized unit. Participants were affected by dementia ranging from mild to severe. The patients were treated with the Reality Orientation Therapy (ROT), integrated, when needed, with individualised cognitive approaches. The results concern: the cognitive status, evaluated by means of the Mini Mental State Examination (MMSE), the functional status, evaluated with the Activity of Daily Living (ADL) scale, the assessment of psychological and behavioural disorders measured with the Neuropsychiatry Inventory (NPI). The cognitive, functional, and psychopathological assessments were administered at admission and discharge. *Results.* The mean MMSE scores at admission and discharge were respectively 16.06 and 17.54 (Wilcoxon Ranks Test: *p* = 0.005). Mean ADL scores were 4.86 at admission and 5.02 at discharge (*p* = 0.011). Mean NPI scores were respectively 21.46 and 12.26 (*p* = < 0.001). *Conclusions.* This survey of the 17-month experience suggests that a comprehensive treatment program may have beneficial effects on cognitive, functional, and in particular neuropsychiatric outcomes. The results should be verified with a randomised clinical trial.

## 1. Introduction

Limited evidence is available that cognitive-behavioural rehabilitation and stimulating activities may delay the clinical onset of dementia [38] and have worthwhile benefits for many adult people with cognitive disorders, especially in association with cholinesterase inhibitors (ChEI) [16,21,24,25,35]. A recent review found that Reality Orientation Therapy (ROT) was associated with significant improvements in both cognition and behaviour [32,33]. On the other hand, it is not at present possible to draw conclusions about the efficacy of other rehabilitation techniques such as validation therapy [22], music therapy [12], and reminiscence therapy [34,39]. Therefore, it is important to collect more evidence about the outcome of non-pharmacological treatments of Alzheimer's Disease (AD), in order to establish management guidelines, as it has been done for the rehabilitation of cognitive functions (language, spatial perception, attention, memory, calculation, praxis), following acquired neurological damage of different aetiology such as stroke

∗Corresponding author: Alberto Raggi, Division of Neurology, Ospedale San Raffaele Turro, Via Stamira d'Ancona 20, 20127 Milan, Italy. Tel.: +39 02 26433305; Fax: +39 02 26433394; E-mail: raggi.alberto@hsr.it.

ISSN 0953-4180/07/$17.00 © 2007 – IOS Press and the authors. All rights reserved and traumatic brain injury [3]. The cardinal principles of a rehabilitative treatment in the elderly [40] may be applied also to patients with cognitive disorders [41]. In contrast to other conditions, rehabilitation in this domain aims mainly at the reduction of the functional impact of disabling conditions, rather than at the achievement of an actual improvement of the neurological condition. The treatment approach should be multidimensional, concerning different professional figures (medical doctors, psychologists, psychotherapists, physical therapists, speech therapists, education specialists, nurses), with the goal to improve as much as possible the quality of life of patients with a chronic disease and that of their caregivers.

The aim of this paper is to report the results of our experience with a comprehensive rehabilitation program in a hospital setting in patients affected by dementia due to probable Alzheimer's disease, on the basis of cognitive, functional, psychological outcomes.

## 2. Methods

### *2.1. Subjects*

Patients with probable AD diagnosed by a senior neurologist before the admission in the ward. Pharmacological therapies with ChEI or other psychotropic medications were started in the months before the beginning of the rehabilitation program and appropriate doses were already established.

### *2.2. Inclusion criteria*

People were considered suitable for full assessment and participation if they: (a) met DSM-IV criteria for dementia due to probable AD (American Psychiatric Association, 1994) [1]; (b) were affected by dementia ranging from mild to severe; (c) had some ability to communicate; (d) were able to hear and see well enough to participate in the group activities and make use of most of the material in the program; (e) did not have major physical illness or disability witch could affect participation; (f) did not have a diagnosis of mental retardation or of a developmental learning disability.

### *2.3. Patients assessment*

A structured medical history collected from the patient and the primary caregiver, a neurological examination, a neuropsychological assessment, routine laboratory analyses and neuroimaging studies had been previously performed, and were consistent with an AD diagnosis [31]. The assessment of the staging of dementia was done with the Clinical Dementia Rating Scale (CDR) [9]. Comorbidity was identified using the Cumulative Illness Rating Scale (CIRS index) [26]. The functional status was evaluated according to the Activity of Daily Living (ADL) [10] and Instrumental Activities of Daily Living (IADL) [15] scales. Cognitive status was evaluated by means of the Mini Mental State Examination (MMSE) [8]. The psychopathological assessment was based on the Neuropsychiatric Inventory (NPI) [5]. The NPI includes a set of screening questions for twelve psychopathological features potentially present in the past four weeks. If a positive response was obtained, then that behavioural domain was explored with standardized questions focusing on specific aspects of that behavioural disturbance. The caregiver then rated the frequency (score range 1–4) and severity (score range 1–3) of the behavioural disturbance. A composite score for each domain was the product of the frequency and severity subscores, with a maximum of 12. Mobility problems were assessed with the Tinetti Scale [37].

### *2.4. Study design and rehabilitation program*

The basal assessment was performed at admission in the ward by a neurologist, a psychologist, an education specialist and a nurse of our staff. Patients were always accompanied by their primary caregiver and some of the tests mentioned above were administered to them. The duration of the rehabilitation program was ratified at the beginning of the hospitalisation and adjusted during the treatment depending from compliance and clinical requirements. Patients with a MMSE score lesser than 10 at admission underwent informal and formal ROT [2] (two sessions of 45 minutes everyday). For those participants with a MMSE score from 10 to 24, ROT was integrated with sessions (once a day) of computerized cognitive training (Cogpack, version 5.9j; Marker Software). The program consists of a series of 30 computer tasks tapping different functional areas at varying levels of difficulty. Some tasks are designed to train attention and concentration, others for verbal, spatial and numerical ability and mem-

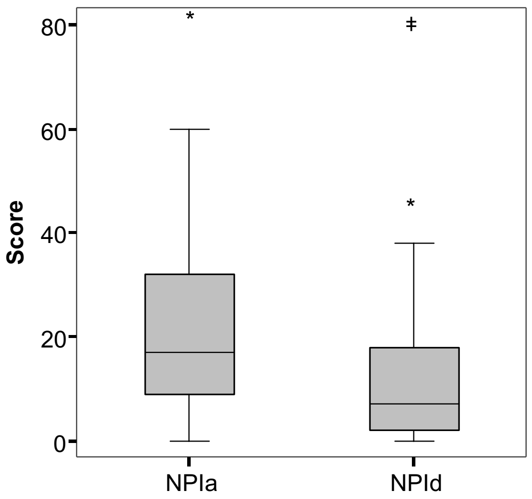

Fig. 1. Boxplot showing Neuropsychiatric Inventory (NPI) scores at admission (NPIa) and discharge (NPId).

ory or psychomotor speed. The tasks are attractively designed, and the level of difficulty is adaptive. Some patients and family members underwent a support psychotherapy. Mobility deficits were treated with physical therapy. The hospitalisation setting was chosen in order to facilitate the admission to the program of subjects with a CDR score of 3, who are usually excluded from standardised rehabilitation treatments. Environmental problems, such as the easy availability of daily transportation from home to hospital have also been taken into consideration while choosing between a day hospital and a full time hospitalisation setting.

### *2.5. Outcome measures*

Cognitive (MMSE), functional (ADL), and psychopathological (NPI) assessments were administered at admission and discharge time in order to assess target outcomes.

### *2.6. Data analysis*

Statistical analyses were performed using SPSS version 12.0.1 software (SPSS, Chicago, IL) and S-Plus 2000 Professional Release 2. Wilcoxon Signed ranks Tests were performed to evaluate the significance of the change in the various scales, as well as the effect of pharmacological treatment at the end of the therapy. This is a nonparametric procedure used to test the hypothesis that the two variables have the same distribution. It makes no assumptions about the shapes of the distributions of the two variables, thus it is appropriate for the considered scales. The statistical test is based on the ranks of the absolute values of the differences between the two variables. To investigate the impact of the disaggregate scores in contributing to NPI a Tukey HSD test has been used. To test whether the proportions of improved patients was more relevant in a CDR class or was constant in all the classes a Chi-Square Proportional test was performed.

## 3. Results

The sample consists of 50 adult patients with probable AD (72 % males, 28 % females). The mean age of the study population was of 76 years (SD: 6.33). Over half of the subjects were aged between 71 and 81 years. The age variable for females shows a higher dispersion than that for males. Extreme values for this variable are in fact ascribed to females subjects. Over half of the sample had five years of education. Mean education was 6.42 years with a SD of 3.66. Mean length of stay in the ward was 26 days (SD: 5.52). Most represented IADL scores were between 1 and 4 (mean: 3.48; SD: 1.89). The score of 4 is the most frequent in our study sample. Most of the subjects show a very low comorbidity: in fact the CIRS index of 50% of them amount to 0 (mean: 0.92; SD: 1.17). Tinetti gait and balance scores were high (mean: 25.80; SD: 4.19). Both CIRS index and Tinetti Scale scores show a low physical impairment. The stage of dementia is distributed in the sample by similar proportions, that is mild (30%), moderate (40%), and severe (30%).

The main analyses concern the outcomes values for the cognitive (MMSE), functional (ADL), and psychopathological (NPI) status. The mean MMSE scores at admission and discharge were respectively 16.06 (SD: 5.60) and 17.54 (SD: 6.45) (Wilcoxon Ranks Test: $p = 0.005$). Mean ADL scores were 4.86 at admission (SD: 1.37) and 5.02 (SD: 1.36) at discharge from the ward (Wilcoxon Ranks Test: $p = 0.011$). Mean NPI scores were respectively 21.46 (SD: 16.68) and 12.26 (SD: 17.28) at the beginning and at the end of the rehabilitation programme (Wilcoxon Ranks Test: $p < 0.001$) (Fig. 1). The contribution of the twelve parameters composing the global NPI outcome scores was analysed. A descriptive analysis suggest that there is a notable improvement of Apathy (corresponding to 50% of the score), Agitation (65%), Delusions (52%) and Hallucinations (62%; low amount of the sample). The Tukey HSD Test allowed to investigate the impact of the disaggregate scores in contributing to NPI. Apathy resulted to be the most influent subgroup. 35 of the participants had been prescribed an acetylcholinesterase inhibitor before the admission in the ward. 13 patients were treated with antidepressants, 12 with benzodiazepines, 14 with antipsychotics. Analysing the effect of each specific drug on the three outcomes, we do not find any significant improvement ($\chi^2$ Proportional Test).

All stages of dementia in our sample seems to be amenable to the hospitalisation treatment, since the proportions are not significantly different in the 3 groups for each analysed index.

## 4. Discussion

A significant effect of the comprehensive treatment program was found on cognitive and functional outcomes. These improvements, observed in a hospitalisation setting, deserve some attention as starting points of a rehabilitation and management intervention to be carried on at a community level. Even more relevant are the outcomes concerning psychopathology.

Neuropsychiatric symptoms are common features of AD [17,19,20], and, in clinical practice, they cause marked distress in patients and caregivers [28] playing a crucial role in the decision to seek long-term institutionalisation [23,36]. Clinical studies show that psychopathological symptoms are amenable to pharmacological treatment, resulting in reduced distress, enhanced quality of life, and extended community stay [6, 11]. A recent review found that ROT was associated with significant improvements in behaviour of patients with dementia [33], but the clinical effectiveness of cognitive-behavioural rehabilitation on neuropsychiatric symptoms is still debated. Our study indicates a significant effect of the treatment program on cognitive and functional outcomes, and a highly significant effect on psychological and behavioural disturbances. In order to describe more specific target outcomes, we have analysed the twelve NPI items separately, finding that the comprehensive management program improves above all apathy, agitation, hallucinations and delusions.

Apathy is among the most common behavioural symptom in subjects with AD [17]. Involvement of the cingulate gyrus is related to symptoms of apathy in AD and other disorders [4,7,18]. Apathy may be attributed in part to lack of environmental stimulation ("exogenous" component of the symptom). Non-pharmacological treatments, such as a kit-based activity intervention and a time and attention control with one-on-one meetings with an activity therapist, seems to reduce significantly this symptom [27]. The authors however suggest that more research is needed to develop specific behavioral interventions for apathy in patients with dementia. The results of our study suggest that environmental stimulations and cognitive-behavioural rehabilitation may contribute to reduce apathy – for the "exogenous" component – and its consequences for patients and caregivers. We attribute this beneficial effect to: continuous stimulation, positive group feeling secondary to the program (ROT) development, and socialization.

Agitation can occur frequently in patients with AD at all stages [30]. It is considered as a manifestation of frontal lobe dysfunction [29]. We have found that cognitive stimulation therapy groups and individual intervention, when needed, may improve this emotional symptom. The support of operators and the limitation of many environmental stressors due to hospitalisation may be helpful in reaching this aim. Indirect effects of reduction of caregiver burden might also contribute to achieve this effect.

It has been suggested that positive symptoms, such as Hallucinations and Delusions, may improve in schizophrenic patients with cognition enhancing interventions systematically addressing motivation and self-esteem [13,14]. This factor, in addition to an adequate pharmacological treatment, may have contributed to the improvement observed in our sample.

In conclusion, the present findings suggest that a global management approach, based on the combination of pharmacological therapy, cognitive-behavioural -motor rehabilitation, and caregivers' support may be useful in dementia, and deserves to be evaluated in a randomised clinical trial. Subjects at all stages of dementia showed some improvement attending the treatment program. The observed improvements need to be reinforced by subsequent day hospital or community programs. In fact it has been shown that a long-term intervention in ChEI-treated AD patients produces additional benefits on neuropsychiatric aspects [24]. The positive effect of the treatment program on the outcomes might be influenced by the fact that our patients, besides the cognitive deficit, had mild mobility impairment and a very low comorbidity assessed with CIRS.
